# Safety and cost-effectiveness of outpatient cervical disc arthroplasty

**DOI:** 10.4103/2152-7806.73803

**Published:** 2010-12-13

**Authors:** Richard Wohns

**Affiliations:** South Sound Neurosurgery, PLLC, 1802 S. Yakima, Tacoma, WA 98405

**Keywords:** Ambulatory surgery, cervical discectomy, cervical vertebrae, cost-effectiveness, disc arthroplasty, spine

## Abstract

**Background:**

To assess the safety, clinical efficacy, and cost-effectiveness of outpatient cervical disc arthroplasty.

**Methods:**

We retrospectively reviewed the records of 26 consecutive patients who underwent outpatient cervical disc arthroplasty between February 2009 and May 2010 in order to assess the safety, clinical efficacy, and cost-effectiveness of the process. Fourteen patients were operated in a —free-standing practice-based ambulatory spine surgery center (MSC) and 12 patients were operated in a hospital-based outpatient surgery center. The mean age of the patient sample was 46 years; 56% were female and 44% were male. Indications for surgery consisted of cervical radiculopathy secondary to single-level soft disc herniation. Charts were reviewed to define patient demographics and medical comorbidities. Operative data, including levels treated, surgery time, time to discharge, and intraoperative complications were collected. Clinical outcomes were collected using the PhDx Clinical Outcomes Database. Need for hospital transfer from the ambulatory surgical center (ASC), emergency room visits, and subsequent hospital admission in the perioperative period were determined from patient records. Complications, patient satisfaction, and outcome were ascertained via review of notes from the first post-operative visit.

**Results:**

There was no mortality and no major complications. Pain was present in 100% and motor deficit in 33% of the patients. There were no co-morbidities reported in the group. There were no cases that required hospital transfer and there were no post-op Emergency Room visits or subsequent hospitalization. At the time of the first post-operative visit, 100% of the patients believed that they were improved and no patient had any post-operative complications. The cost of outpatient single-level cervical disc arthroplasty was 62% less than the outpatient single-level cervical anterior discectomy with fusion using allograft and plate and 84% less than the inpatient single-level cervical disc arthroplasty.

**Conclusions:**

Outpatient cervical disc arthroplasty is safe and clinically efficacious in selected patients and is cost-effective compared with both inpatient cervical disc arthroplasty and outpatient anterior discectomy with fusion.

## INTRODUCTION

Overnight hospital stay has been the rule for patients undergoing anterior cervical discectomy and fusion (ACDF) and cervical disc arthroplasty. However, ambulatory surgery has been used with increasing frequency for the treatment of patients with lumbar disc herniations.[1] Similarly, ambulatory surgery for ACDF has been utilized with increasing frequency and has been demonstrated to be safe and efficacious.[5,16] The known complications of ACDF surgery[6] have not been reported to be of any higher frequency in the outpatient setting, and actually are indeed lower.[5]

To determine the safety and efficacy of outpatient ACDF in an outpatient setting, we reviewed our experience over a 15-month period with outpatient cervical disc arthroplasty. The procedure is performed in the same manner as when performed in an inpatient setting and, therefore, our review has focused on the aspects of our experience that are pertinent to the ambulatory setting rather than the long-term efficacy of the procedure, which has been reported elsewhere.[8]

To determine the cost-effectiveness, we compared the entire cost of care for outpatient cervical disc arthroplasty with inpatient cervical disc arthroplasty and with outpatient anterior cervical discectomy with fusion using allograft and plate.

## MATERIALS AND METHODS

A prospectively maintained database, PhDx Clinical Outcomes Database, was utilized to identify all patients who underwent outpatient cervical disc arthroplasty either in our practice’s outpatient spine surgery center (MSC) or in the outpatient operating room at a local hospital, between February and October 2009. Twenty-six patients were identified, 14 of whom underwent disc arthroplasty in the MSC and the other 12 in the hospital’s outpatient surgery center. The outcome data were then reviewed retrospectively. Excluded from outpatient surgery were patients with significant cardiac or pulmonary problems, poorly controlled diabetics, patients weighing over 300 pounds, and those with significant myelopathy. Records from the MSC were reviewed for operative data, operative time, time from completion of surgery to discharge, need for transfer to an inpatient facility, and intraoperative complications.

Patient charts, including clinic visits, correspondence, and hospital reports, were also reviewed. History and physicals were reviewed to obtain demographic information, including age, sex, medical comorbidities, and indications for surgery.

Need for Emergency Room (ER) visits or hospital admission subsequent to discharge from the ambulatory surgical center (ASC) was also assessed via the charts. Records from the first post-operative visit were reviewed to assess perioperative complications and short-term outcome.

Explanations of benefits (EOBs) were reviewed and the average cost for an outpatient cervical disc arthroplasty was calculated. The cost includes billed charges for the technical component, implant, and professional fee. We compared this average cost with pooled data reflecting the average billed charges for the same procedure performed on an inpatient basis and also compared this data with the average cost for an outpatient anterior cervical discectomy with fusion.

## RESULTS

Of the 26 patients included in this study, the mean the mean age was 46 years, with a slight female predominance (56%). None of the patients reported any comorbidities.

All patients (100%) had neck and arm pain, and many (44%) had an objective or subjective sensory disturbance [Table 1]. Motor deficit was present in 33% of the patients, as listed in Table 1, and was categorized based on the weakest muscle group. All 26 patients underwent single-level surgery with the Synthes ProDisc-C arthroplasty device. The average operative time was 40 min and the average recovery time prior to discharge was 3 h. A minimum 3-h stay in recovery had been previously instituted for outpatient ACDF cases.[5]

**Table 1:** Pre-operative Symptoms

Patient conditions	Pre-op %n
Persons reported	9
Neck pain	100.0
Arm pain	100.0
Bowel/bladder dysfunction	0.0
Motor deficit	33.3
Sensory deficit	44.4
Reflex deficit	22.2
Gait disturbance	0.0
Fine motor movement diff.	0.0
None	0.0

There were no transfers to a hospital, no post-operative ER visits, and no late hospitalizations.

All patients presented for their first post-operative visit at an average of 21 days after surgery. One hundred percent of the patients reported improved symptoms at the first visit and there were no patients who felt that their symptoms had worsened. There were no major complications, including no hematomas, dysphagia, hoarseness, vocal cord paralysis, or infections. There were no cases of neurological worsening or persistent pain.

The average EOB charges for a one-level outpatient cervical disc arthroplasty were $11,144.83. The average charges for a one-level outpatient anterior cervical discectomy with fusion were $29,313.43. The average charges for a one-level inpatient cervical disc arthroplasty were $68,000. The average charges for a one-level inpatient anterior cervical discectomy with fusion were $61,095.49.

## DISCUSSION

In the 20^th^ century, there was a gradual trend toward ambulatory surgery, from early reports of outpatient surgery in the 1900s to the development of free-standing ambulatory surgery centers in the 1970s.[18] Many procedures that were once carried out only on an inpatient basis were moved to an outpatient setting, and this shift was associated with high levels of patient and physician satisfaction as well as a decrease in the cost.[2,7,9,14] Outpatient spine surgery was reported as early as 1987[11] for the lumbar spine and 1996[12] for anterior cervical spine surgery, but the numbers of patients treated were small.

Patient safety has been the prime issue in the transition of anterior cervical spine surgery to an outpatient setting. Of particular concern are the possibilities of airway compromise from post-operative hematoma or soft tissue swelling in the immediate post-operative period, epidural spinal cord compression by post-operative hematoma, or cerebrospinal fluid fistula. Inpatient ACDF series have reported a wide range of incidences of post-operative hematoma, with 2.4% of the patients requiring evacuation of a hematoma in a recent series of 1015 patients.[4] Another recent inpatient series concluded that outpatient ACDF would be safe as all of the post-operative hematomas in their experience were found within 6 h of surgery.[17] There have been no airway problems or major complications in the previously reported series of outpatient ACDF,[3,5,13,16,17] the largest of which had 390 patients.

Three patients in our series required treatment for nausea and vomiting. This reaction to anesthesia is not uncommon, and is a concern with any type of ambulatory surgery. In order to reduce the incidence of this occurrence, patients are now treated prophylactically with famotidine, ondansetron, and dexamethasone and the use of opioids is minimized.

Increased neurologic deficit and worsening of myelopathy have been described after ACDF.[4] In our series, there were no such cases.

Dysphonia and dysphagia are common after anterior cervical spine surgery. When these symptoms are studied systematically, their incidence has been found to be higher than is typically reported in case series.[10,15] In retrospective reviews, such as the present one, whether these complaints are reported will be influenced by the length of time to follow-up and whether the patient finds them troublesome enough to report and whether the physician finds them significant enough to record. With respect to performing the procedure on an outpatient basis, it is important to verify that all patients are able to swallow at least liquids before discharge. ER visits or admission for dysphagia did not occur in our series.

Patient selection is obviously an important determinant in the safety of outpatient spine surgery. While we do not have an absolute criteria for exclusion, we have generally treated relatively healthy patients at our ASC. Patients older than 65 were excluded from this series.

Myelopathy is another important consideration in patient selection, particularly when there is pre-operative gait impairment, which may make early ambulation and discharge more difficult. None of the patients in our series had myelopathy or any gait impairment.

Ultimately, the decision to proceed with outpatient surgery is made in conjunction with the patient, after thorough discussion of the options. Patients with anxiety about ambulatory surgery, significant pre-operative narcotic use, low pain threshold, and those who may have a difficult time caring for themselves may be better served with surgery in a hospital.

Our experience reflects on the safety and efficacy of outpatient cervical disc arthroplasty in a select group of patients, excluding those who are over 65 years old and those with significant medical comorbidities.

Cost-effectiveness for one-level outpatient cervical disc arthroplasty was demonstrated. Compared with one-level outpatient anterior cervical discectomy and fusion, the disc arthroplasty procedure was approximately 62% less costly and compared with the one-level inpatient cervical disc arthroplasty, was 84% less expensive.
